# Groundwater Potential Mapping Combining Artificial Neural Network and Real AdaBoost Ensemble Technique: The DakNong Province Case-study, Vietnam

**DOI:** 10.3390/ijerph17072473

**Published:** 2020-04-04

**Authors:** Phong Tung Nguyen, Duong Hai Ha, Abolfazl Jaafari, Huu Duy Nguyen, Tran Van Phong, Nadhir Al-Ansari, Indra Prakash, Hiep Van Le, Binh Thai Pham

**Affiliations:** 1 Vietnam Academy for Water Resources, Hanoi 100000, Vietnam; 2 Institute for Water and Environment, Hanoi 100000, Vietnam; hahaiduongcwe@yahoo.com; 3 Research Institute of Forests and Rangelands, Agricultural Research, Education, and Extension Organization (AREEO), P.O. Box 64414-356 Tehran, Iran; ajaafari@gmail.com; 4 Faculty of Geography, VNU University of Science, Vietnam National University, 334 Nguyen Trai, Hanoi 100000, Vietnam; huuduy151189@gmail.com; 5 Institute of Geological Sciences, Vietnam Academy of Sciences and Technology, 84 Chua Lang Street, Dong da, Hanoi 100000, Vietnam; tphong1617@gmail.com; 6 Department of Civil, Environmental and Natural Resources Engineering, Lulea University of Technology, 971 87 Lulea, Sweden; 7 Department of Science & Technology, Bhaskarcharya Institute for Space Applications and Geo-Informatics (BISAG), Government of Gujarat, Gandhinagar 382002, India; indra52prakash@gmail.com; 8 Institute of Research and Development, Duy Tan University, Da Nang 550000, Vietnam; 9 University of Transport Technology, Hanoi 100000, Vietnam; binhpt@utt.edu.vn

**Keywords:** groundwater potential mapping, ensemble modeling, spatial modeling, machine learning

## Abstract

The main aim of this study is to assess groundwater potential of the DakNong province, Vietnam, using an advanced ensemble machine learning model (RABANN) that integrates Artificial Neural Networks (ANN) with RealAdaBoost (RAB) ensemble technique. For this study, twelve conditioning factors and wells yield data was used to create the training and testing datasets for the development and validation of the ensemble RABANN model. Area Under the Receiver Operating Characteristic (ROC) curve (AUC) and several statistical performance measures were used to validate and compare performance of the ensemble RABANN model with the single ANN model. Results of the model studies showed that both models performed well in the training phase of assessing groundwater potential (AUC ≥ 0.7), whereas the ensemble model (AUC = 0.776) outperformed the single ANN model (AUC = 0.699) in the validation phase. This demonstrated that the RAB ensemble technique was successful in improving the performance of the single ANN model. By making minor adjustment in the input data, the ensemble developed model can be adapted for groundwater potential mapping of other regions and countries toward more efficient water resource management. The present study would be helpful in improving the groundwater condition of the area thus in solving water borne disease related health problem of the population.

## 1. Introduction

Groundwater is one of the major natural resources due to its importance for residential, agricultural, and industrial water supply [1,2,3]. With the rapid population growth, industrial development, and increased domestic use, most of the countries of the world will face the fresh water shortage problem by 2025 [4]. Economic and demographic developments in the world in general and in Vietnam, in particular, are causing ever-increasing water demands [5]. Given the increased demand for water for various purposes (e.g., agriculture, industry, and human consumption), most of the groundwater water reservoirs have been over-exploited [6]. Thus, identifying areas with high groundwater storage potential is important for effective water resource management.

Groundwater potential refers to the possibility of groundwater occurrence or the amount of groundwater storage across an area [7,8]. Over the past few years, many efforts have been made to assess the groundwater potential in different regions of the world by different researchers [7,9,10]. In these studies, Geographic Information Systems (GIS) and remote sensing-based approaches have been effectively applied for mapping of groundwater potential. However, the models used in these studies are based on expert opinion or traditional weighted methods; thus, the effectiveness of the assessment of groundwater potential was subjective and not adequately accurate.

In recent years, with the help of advance information technology, machine learning has been introduced and applied to solve a lot of real-world problems including groundwater potential mapping [11]. Recently Pal et al. [12] applied the machine learning methods namely Random Forest (RF), Radial Basis Function Classifier (RBFC) and Artificial Neural Network (ANN) to assess the capacity of the groundwater potential in the Tangon watershed in eastern Indian and Bangladesh. Naghibi et al. [13] applied the Boosted Regression Tree (BRT), Classification And Regression Tree (CART), and RF model to map the groundwater potential in the Koohrang Watershed of Iran.

More recently, different hybrid ensemble machine learning models which combine a base model with the optimization algorithms or ensemble techniques have been proposed for achieving higher reliability in groundwater potential mapping. Miraki et al. [14] developed an ensemble model (RS-RF) using a combination of RF and Random Subspace ensemble technique to assess the groundwater potential in the Qorveh-Dehgolan plain, Kurdistan province, Iran, and reported that the RS-RF model is a promising tool for mapping of groundwater potential. Al-Fugara et al. [15] combined Support Vector Machine (SVM) and Genetic Algorithm (GA) to build a hybrid model for mapping groundwater potential in the Jerash and Ajloun region of Jordan. Naghibi et al. [16] used Adaboost, Bagging, Generalized Additive to optimize Naïve Bayes for better performance of groundwater potential modeling. In recent study, Banadkooki et al. [17] proposed to use the whale optimization algorithm for optimizing a base ANN model for groundwater potential mapping and demonstrated the enhanced predictive performance of the hybrid model.

In general, machine learning methods and their derived hybrid and ensembles models are promising for the development of reliable groundwater potential maps. Therefore, in this study, the main aim is to assess the groundwater potential using a hybrid model (RABANN) which is a combination of the ANN—a popular machine learning model, and an ensemble technique, namely RealAdaBoost (RAB). The main difference of this study compared with previous published works is that this is the first time an ensemble classifier framework of the RAB and the ANN was constructed to improve the performance of groundwater potential mapping. With this objective, the DakNong province of Vietnam was selected as the study area where groundwater problem exists and sufficient hydrology and geo-environmental data is available, and where no advanced modeling technique and approach was applied to assess groundwater potential. Area Under the Receiver Operating Characteristic (ROC) curve (AUC) and several statistical performance measures were used to validate and compare performance of the ensemble RABANN model with the single ANN model. Weka open source software and standard GIS software were used for the development of the models and visualization of the potential maps, respectively.

## 2. Study Area

**Figure 1:**
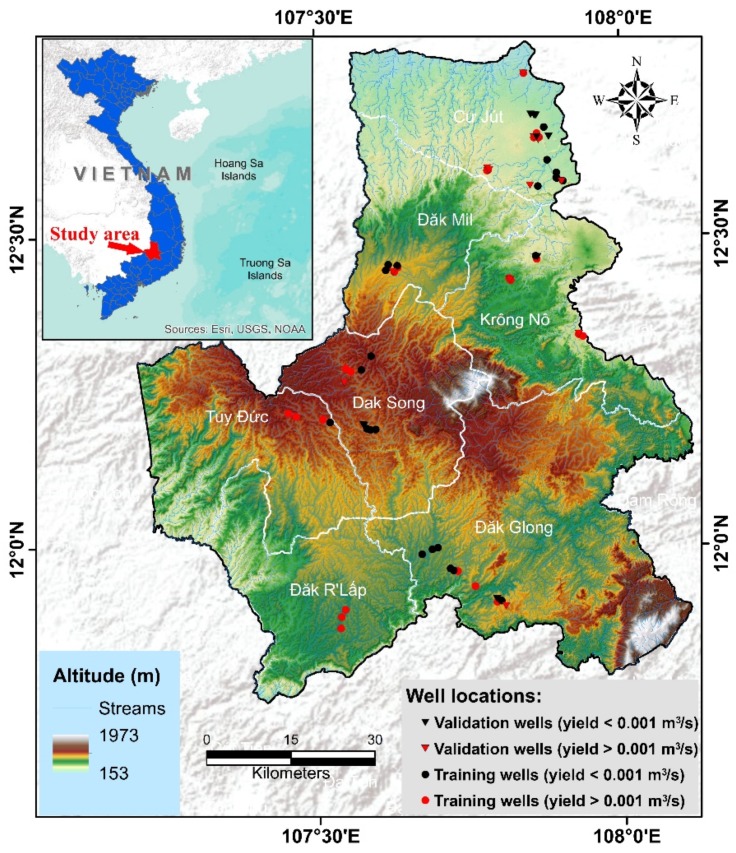
Location map of the study area.

The DakNong province (11°45′ to 12°50′ N latitude, 107°13′ to 108°10′ E longitude) is a transitional area between the two sub-regions of the central highlands and the southeast part of Vietnam (Figure 1). This province has an average elevation of about 650m above mean sea level, some places have higher elevation up to 1982 m. This province has a diverse topography, strongly divided with high mountains with large, sloping, fairly flat highlands alternating with low-lying plains. Low valley topography, with a slope of 0–3° mainly distributed along the Krong No and Serepok rivers. The plateau terrain has an average elevation of 700 m, with a slope of about 5–10°.

The climate regimes having the common characteristics of the tropical equatorial monsoon climate. Each year has two distinct seasons: the rainy season from April to the end of November, concentrating over 90% of the annual rainfall; dry season from December to the end of March next year, the rainfall is insignificant. The annual average temperature is 22–23 °C, the highest temperature is 35 °C. The average annual rainfall is 2513 mm. The precipitation mainly concentrates in the month of August and September.

**Figure 2:**
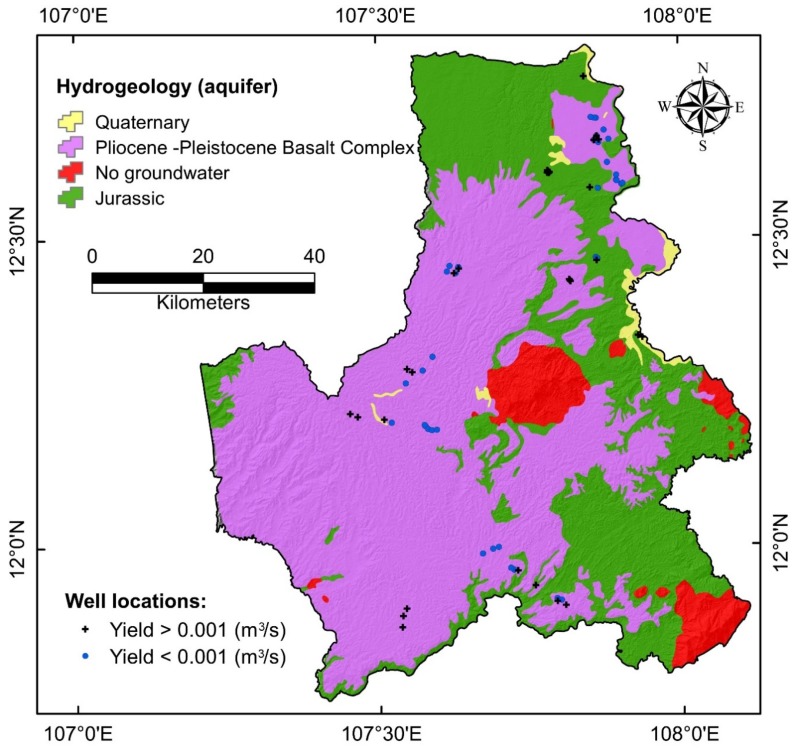
Hydrogeological map of the study area.

Hydro-geologically, there are three main types of aquifer presenting in Dal Lak province (Figure 2) namely Quaternary, Pliocene—Pleistocene Basalt Complex, and Jurassic:

(i) Quaternary aquifer comprises of alluvium (gravel, pebbles, grit, sand, clay) along the main rivers and large streams with an area of about 27.16 km^2^. Its thickness varies from 5 to 20 m, average 5 to 7 m. This aquifer is of unconfined type. Water depth varies from 0.0 to 10.7 m, average 2 to 4 m. Water levels fluctuate almost in phase with the fluctuating cycle of rainfall. In general, the level of water richness of this aquifer is classified as poor to medium. In many places, the water dried up during the dry season. This shows that the groundwater of the aquifer is limited and can only be exploited for residential areas on a small scale.

(ii) Pliocene—Pleistocene Basalt complex aquifer comprises of different types of basalt rocks (weathered, dense and vesicular) and occupies about 3936.53 km^2^ area. Its thickness varies from 27 to 502 m, the average thickness is about 100 m. Water flows in the basaltic complex through joints, cracks, interconnected vesicles and cavities and also through weathered rock zones. Thickness of permeable zones forming aquifer varies from 20 to 100 m. Groundwater occurs in unconfined condition. In general, this aquifer has average water permeability and storage; and water is of good quality.

(iii) Jurassic aquifer occupies an area of about 2116.78 km^2^. Lithological composition of this aquifer is mainly sandstone, siltstone, limestone, and schist. The thickness of the aquifer varies from 17.5 to 79.6 m, average 40 m. Water exists in the form of fissures—seams and is often discontinuous. Regarding hydraulic properties, water is of non-pressurized type (unconfined aquifer), sometimes with local pressure. In general, this aquifer is widely distributed, but the level of permeability and water content is poor, not uniform.

## 3. Materials and Methods

### 3.1. Data Used

**Figure 3:**
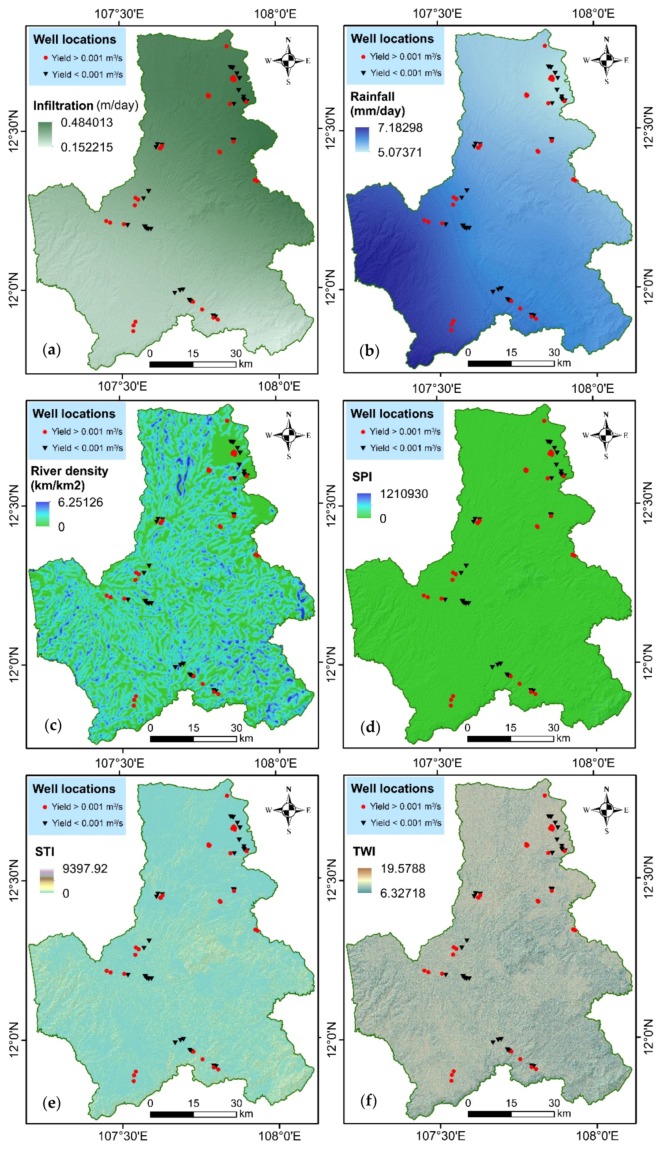

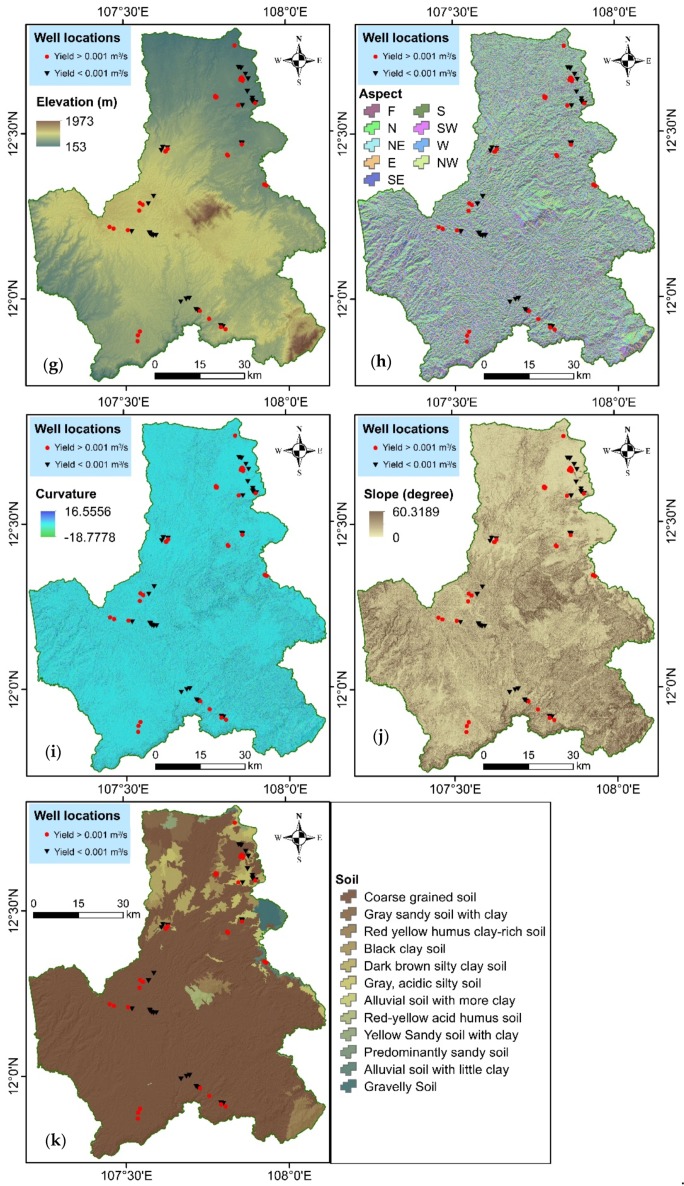

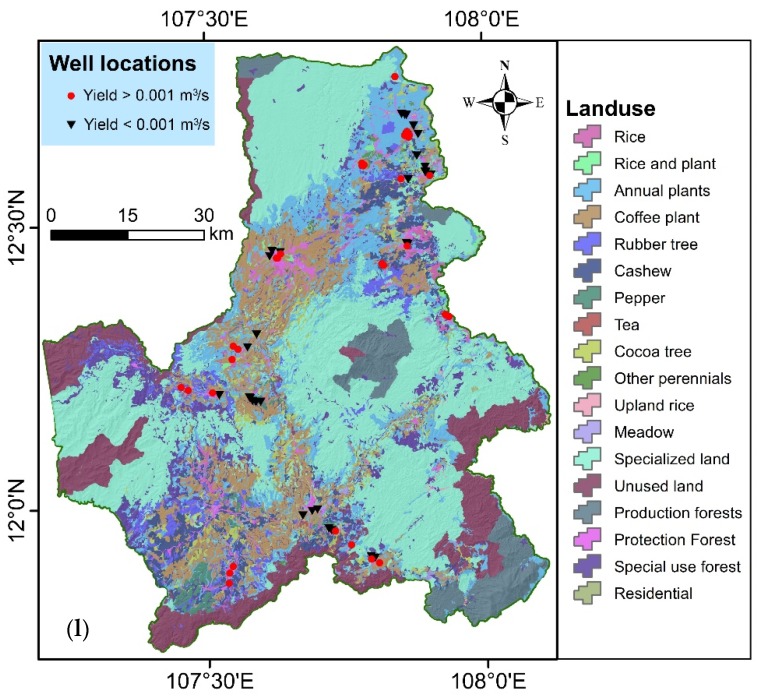
Maps of groundwater influencing factors: (**a**) infiltration, (**b**) rainfall, (**c**) river density, (**d**) Stream Power Index (SPI), (**e**) Sediment Transport Index (STI), (**f**) Topography Wetness Index (TWI), (**g**) elevation, (**h**) aspect, (**i**) curvature, (**j**) slope, (**k**) soil, and (**l**) land use.

Sub-surface and surface data is required for assessing groundwater potential of an area [18,19]. In this study, in total 72 wells groundwater data including yield data was used in conjunction with twelve groundwater potential influencing factors, namely infiltration, rainfall, river density, Stream Power Index (SPI), Sediment Transport Index (STI), Topographic Wetness Index (TWI), elevation, aspect, slope, curvature, soil, and land use were used. A 30-m resolution Digital Elevation Model (DEM) collected from United States Geological Survey (https://earthexplorer.usgs.gov) was used for the construction of topographical (i.e., elevation, aspect, slope, and curvature) and hydrological (SPI, STI, and TWI) maps. Land use map (1:100,000) was collected from the DakNong Department of Natural Resources and Environment. Geology (1:100,000) and average daily rainfall maps were obtained from the hydrogeological map of South Central and Central Highland Vietnam. More detail, topography is very important as groundwater table generally follows surface topography. Run-off flows from higher elevation to lower elevation, therefore, elevation is considered as one of the most important factors in groundwater potential mapping. Curvature of the ground is important as concave surface are more suitable for holding the surface water thus helps in recharging the area. Aspect give direction of slope and thus provide information of incidence of rainfall [20,21,22,23]. Slope provide important information of runoff and accumulation of water thus of recharge. The slope has the tendency of inverse proportionality with the groundwater potential [24]. TWI presents the topography-hydrology relationships of the landscape, and is typically used to quantify topographical control on hydrological process [25,26]. SPI and STI describe erosive processes that are caused by surface runoff and are proxies for the intermediate scale topographic position (ridge, slope, or valley bottom) and the stream capacity of the landscape [27,28]. In general, the regions with higher SPI and STI values have higher potential for groundwater occurrence because they have higher water table [29]. River density presents the drainage capacity which is inverse proportionality of the soil infiltration [10,30,31]. Rainfall is one of the most important factors in groundwater potential model because the more precipitation region are likely to have more groundwater potential [9]. Opposite to rainfall, river density has a reverse relationship with groundwater potential [32] because when the drainage density is lower, the infiltration and recharge are greater [29]. Land use presents the influence of human activities on the landscape evolutions [33,34,35]. Soil type indicates the filtration rate and, therefore, is another important factor for groundwater potential [36,37]. The maps for the groundwater influencing factors are shown in Figure 3.

### 3.2. Methods Used

#### 3.2.1. Artificial Neural Networks

Artificial Neural Networks (ANN) is one of the efficient modeling techniques for finding the hidden patterns from data by mimicking human brain action. The ANN enables the transmission of information from one multivariable space to another multivariable space [38]. It is a widely used approach for pattern recognition and classification problems [39,40,41]. The data statistical distribution is independently performed by the ANN and specific statistical parameters are not required for obtaining the estimation results. This model is a universal approximator that performs parallel processing of the information from the data to approximate a large class of functions with a high degree of accuracy. This method utilizes the characteristics of the data for the procedure and, therefore, avoids any prior assumption in the model building. The ANN is a three-layered network connected by acyclic links. The input-output relationship in the ANN can be given as follows [42]:

(1) $$y_{t}=w_{0}+\sum_{j=1}^{q}\omega j_{}._{}g(w_{0,j}+{\sum_{i=1}^{p}w_{i,j}}_{}._{}y_{t-i})+z_{t}$$

where *y_t_* is output, *y*_*t*−*i*_ is input, and *w*_*i*,*j*_ (i = 0, 1, 2,…, *p*, *j* = 0, 1, 2,…, *q*) and *w_j_* (*j* = 0, 1, 2,…, *q*) are the model parameter, *p* is the number of input nodes, and *q* is the number of hidden nodes.

The ability to process large datasets and achieving accurate estimations using small training data are the main advantages of the ANN. Fundamentals of the ANN and reviews of its applications can be found in the literature [43,44,45].

#### 3.2.2. RealAdaBoost

RealAdaBoost (RAB) is an ensemble learning technique algorithm developed in 1999. In this algorithm, two discrete values are grouped together on the output of continuous confidence [46]. It uses repeated execution of weak learning algorithms by calling it to find a small number of weak classifiers and then combining them into a strong one with the objectives of partition determinations on all the data to raise the accuracy of any learning model [47]. In this algorithm, the weak classification was evaluated confidence using the map from space to space with real value instead of the Boolean prediction [48]. The proven advantages of the RAB ensemble technique motivated us to use this technique in combination with the ANN for developing the ensemble RABANN model for groundwater potential mapping.

#### 3.2.3. Validation Methods

Validation performance is a critical step in a modeling procedure, for which several statistical indices has been suggested and used [13,14,49,50,51,52]. In this study, we used Area Under Receiver Operating Characteristic (ROC) curve (AUC) [39,53,54,55,56], Root Mean Squared Error (RMSE) [57,58,59,60,61,62,63,64], Kappa, Accuracy (ACC), Specificity (SPF), Sensitivity (SST), Negative predictive value (NPV), and Positive predictive value (PPV) [65,66,67,68,69]. Detail description of these indices is presented in published literature [61,70,71,72,73,74,75,76,77]. In general, lower RMSE and higher values of AUC, Kappa, ACC, SPF, SST, NPV, and PPV indicate higher model performance [57,58,65,78,79,80,81,82]. Mathematically, these performance indices are given by [60,77,83,84,85,86,87]:

(2) $$\mathrm{PPV}=\frac{\mathrm{TP}}{\mathrm{TP}+\mathrm{FP}}$$

(3) $$\mathrm{NPV}=\frac{\mathrm{TN}}{\mathrm{TN}+\mathrm{FN}}$$

(4) $$\mathrm{SST}=\frac{\mathrm{TP}}{\mathrm{TP}+\mathrm{FN}}$$

(5) $$\mathrm{SPE}=\frac{\mathrm{TN}}{\mathrm{TN}+\mathrm{FP}}$$

(6) $$\mathrm{ACC}=\frac{\mathrm{TP}+\mathrm{TN}}{\mathrm{TP}+\mathrm{FN}+\mathrm{FP}+\mathrm{TN}}$$

(7) $$\mathrm{Kappa}=\frac{P_{\mathrm{obs}}-P_{\exp}}{1-P_{\exp}}$$

(8) $$\mathrm{AUC}=\frac{\left(\sum \mathrm{TC}+\sum \mathrm{TD}\right)}{\left(A+B\right)}$$

(9) $$\mathrm{RMSE}=\sqrt{\frac{1}{N}\sum_{i=1}^{N}{\left(y_{i}-{\bar{y}}_{i}\right)}^{2}}$$

where TP is true positive, TN is true negative FP is false positive, FN is false negative, TC is the number of correctly classified pixels, TD is the number of incorrectly classified pixels, A is the total number of groundwater pixels, B is the total number of non-groundwater pixels, N is the number of samples in the dataset, *y_i_* is the predicted value of the *i^th^* sample, and ${\bar{y}}_{i}$ is the measured value of the *i^th^* sample.

#### 3.2.4. Modeling Methodology

**Figure 4:**
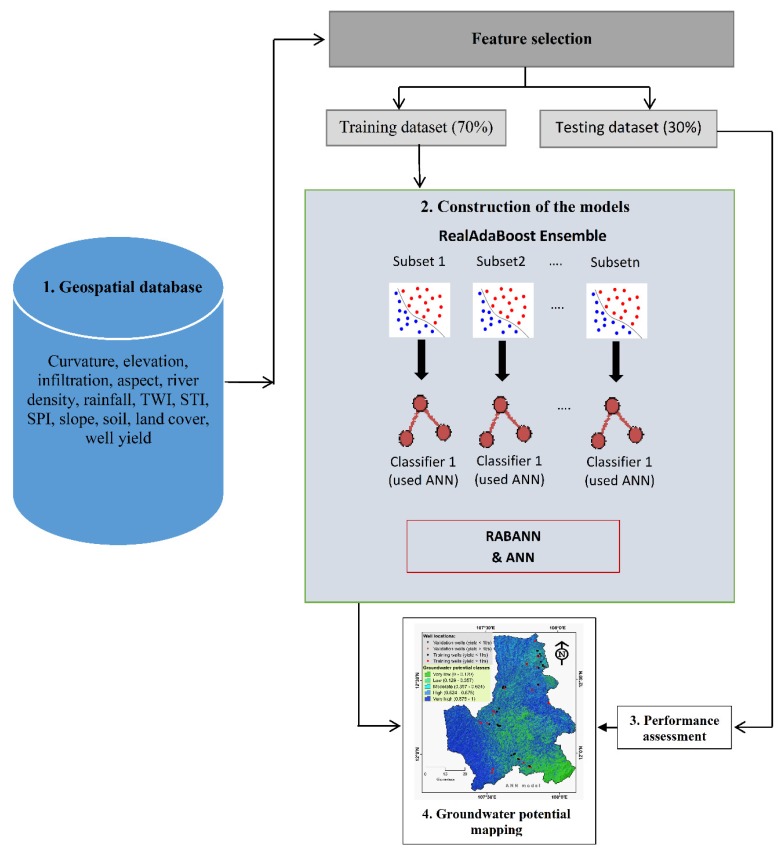
Flowchart of the modeling methodology (Wherein: SPI, stream power index; STI, sediment transport index; topography wetness index, TWI, ANN, artificial neural network; and RABANN, the ensemble model of RAB and ANN).

Groundwater potential models were developed in four main steps (Figure 4): (1) Collection of data from various sources (e.g., available literature, government, and field survey), (2) Development of the models, (3) Validation of the models, and (4) Generation and analysis of the groundwater potential maps. The main step was the development of the models that was conducted in several phases. We first randomly divided the well data (72 locations) into two sets such that one set with 70% of locations (~50) was used for training the models and the remaining locations (~30% = 22 locations) were used for the validation [34,74,88,89,90]. Regarding the set of influencing factors, we used correlation-based feature selection method [91] to measure the average merit of each factor for mapping the groundwater potential. We next overlaid the training and validations datasets with each one of the influencing factors to extract the factor values for generating the final training and validations datasets [92,93,94]. Using these datasets, groundwater potential mapping was formulated as a binary classification procedure, in which the goal was to distinguish between potential and non-potential groundwater classes. Well yield of 0.001 m^3^/s was used as a threshold value to separate non-potential groundwater classes. Finally, the two predictive models based on the ANN and RAB techniques were developed: the single ANN model and the ensemble RABANN model. The single ANN model was constructed with twelve input layers, ten nodes in the hidden layer, and two output layers. Using the ANN as a base model, the ensemble RABANN model was developed where RAB was used as a learning technique to optimize the training dataset used for training the base ANN model.

After successful training the two models, they were validated and compared using the validation methods described in Section 3.2.3. In the final step of modeling methodology, the maps for the groundwater potential of the study were produced and classified into very high, high, moderate, low, and very low potential classes.

## 4. Results and Discussion 

### 4.1. Factor Importance

Quantifying the importance of the twelve influencing factors using the correlation-based feature selection method [91] ranked the factors in terms of their average merit (AM) and revealed that elevation, SPI, STI, river density, aspect, and infiltration with AM >0.1 are the most important factors for the development of groundwater models for our study area (Table 1). Although not very significant, the other factors with AM <1 can be useful for developing the models. Therefore, we opted to use all twelve factors for the modeling process. An examination of the corresponding literature reveals that influencing factors for groundwater potential mapping are area-specific and cannot be exactly compared with other regions. For example, Bui et al. [29] identified TWI, distance from rivers, and SPI as the most important factors for groundwater potential in the Chilgazi watershed, Iran, whereas Chen et al. [95] reported that lithology, elevation, and SPI were the factors that contributed the most to groundwater potential in the Ningtiaota region, China. In contrast to Bui et al. [29], Chen et al. [95], and our results, Kalantar et al. [96] reported on the disadvantage of SPI for groundwater potential modeling in the Haraz watershed, Iran. These differences call for additional research for identifying factors that contribute the most to groundwater potential modeling and mapping in different regions.

### 4.2. Model Performance

Based on application of different statistical indices, the single ANN model and the ensemble RABANN were validated and compared for recognizing the general pattern of groundwater potential (i.e., training performance) and predicting future groundwater occurrences in the study area. In the training phase, the RABANN achieved the highest values of TP (26), TN (27), PPV (96.30%), NPV (93.10%), SST (92.86%), SPF (96.43%), ACC (94.64%), and Kappa (0.893) indices, and lowest FP (1), FN (2), and RMSE (0.224) (Table 2). These results revealed that the ensemble RABANN model correctly classified 93.1% of all pixels in potential class, classified 95.2% of all pixels in non-potential class, classified 92.86% of groundwater pixels into the potential class, classified 96.43% of non-groundwater pixels in the non-potential class, classified ~94.64% of all training pixels, with a perfect (Kappa = 0.893) agreement between predicted and observed well locations.

In the case of predicting future groundwater occurrences (i.e., validation performance), once again our ensemble model outperformed the single ANN model by achieving the highest values of TP (8), TN (8), PPV (61.54%), NPV (72.73%), SST (72.73%), SPF (61.54%), ACC (66.67%), and Kappa (0.338) indices, and lowest FP (5), FN (3), and RMSE (0.504) (Table 2).

**Figure 5:**
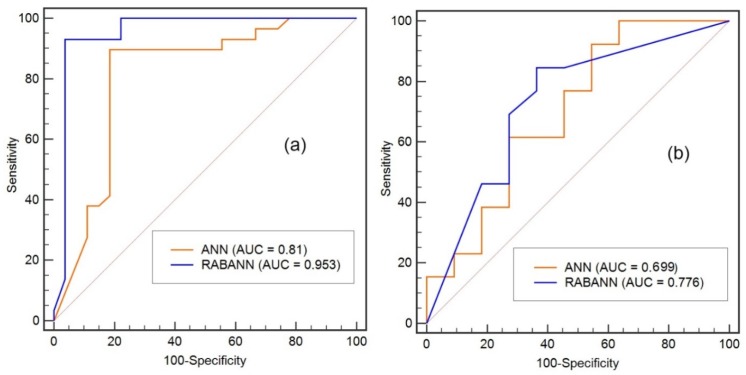
Receiver Operating Characteristic (ROC) curves and AUC values of the models: (**a**) training and (**b**) validation datasets.

The ROC methods further demonstrated that the ensemble RABANN model have higher training (AUC = 0.953 vs. AUC = 0.81) and validation (AUC = 0.776 vs. AUC = 0.699) performances than the single ANN model (Figure 5).

Overall, our results show that the RAB ensemble techniques performed well in improving the performance of the base ANN model. These results are in line with previous works that demonstrated the advantages of ensemble modeling approaches over single simple modeling. For example, J48 decision tree integrated with Bagging [97] and Naïve Bayes tree integrated with Random Subspace [98] for landslide prediction, RF integrated with different ensemble techniques for gully erosion [31], and alternating decision tree integrated with AdaBoost [29], fisher’s linear discriminant function integrated with Bagging [99], RF integrated with Random Subspace [14], and decision stump with different ensemble techniques for groundwater potential mapping [100].

### 4.3. Groundwater Potential Mapping

**Figure 6:**
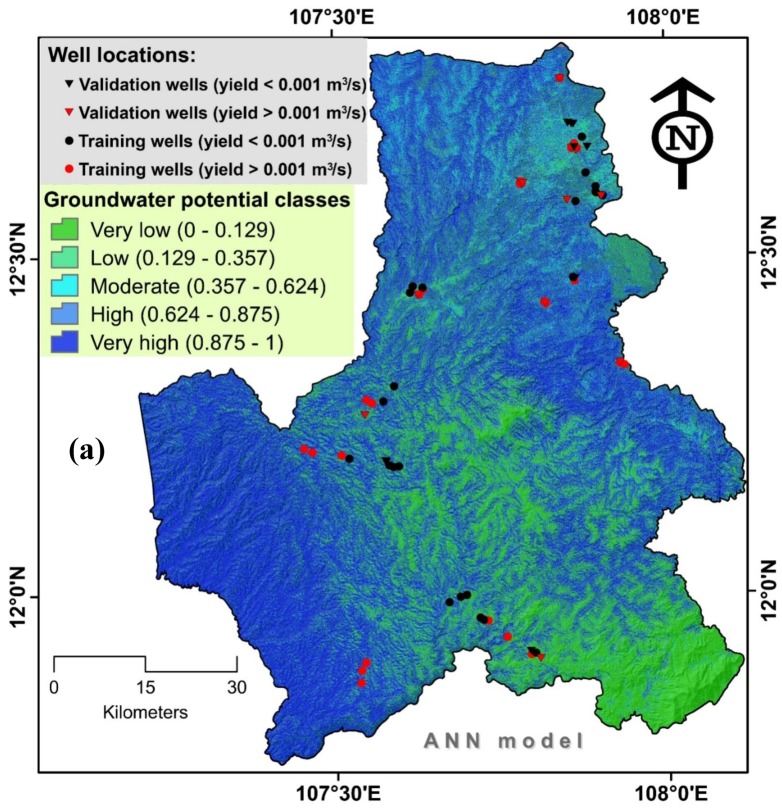

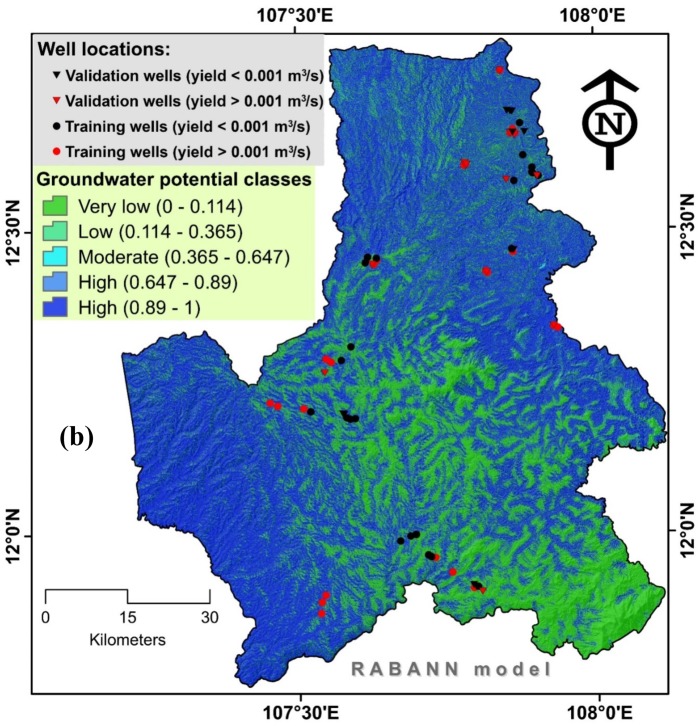
Groundwater potential maps produced using (**a**) ANN (Artificial Neural Networks) and (**b**) RABANN (the ensemble model of RAB and ANN).

**Figure 7:**
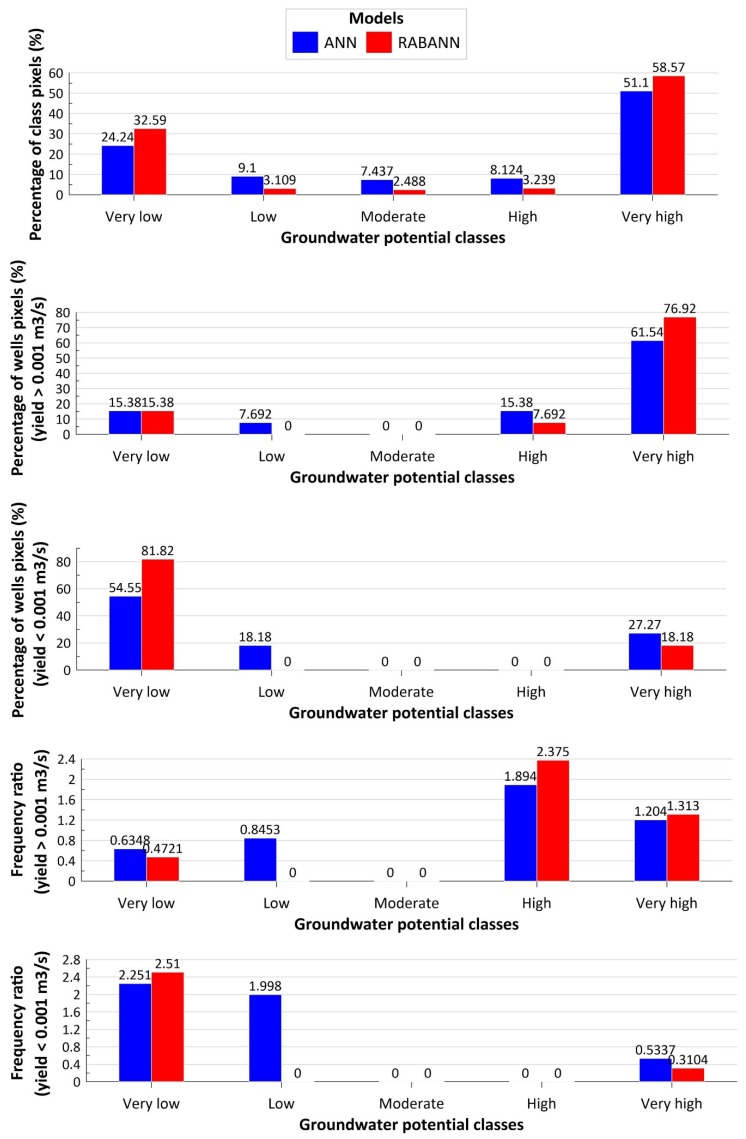
Analysis of the groundwater potential maps.

The ultimate outcomes of the single ANN and ensemble RABANN models were generation of two groundwater potential maps (Figure 6). These maps were classified into five classes: very low, low, moderate, high, and very high potential for groundwater occurrences. Reliability analysis of the maps was carried out using frequency ratio and showed that most of high yield well locations were observed in very high groundwater potential classes, indicating that the models performed well in classifying the study area with respect to well locations (Figure 7).

## 5. Conclusions

Determination of the area with high groundwater potential is one of the important steps in land use planning and water resource management. Up to now, there was no systematic effective scientific study to evaluate groundwater potential of the DakNong province. Therefore, we addressed this gap and developed an ensemble modeling approach to achieve the most accurate and reliable estimate of groundwater potential of this province. In this study, we used an advanced ensemble machine learning model (RABANN) that integrates ANN with the RAB ensemble technique. Apart from providing a distribution map of groundwater potential for the study area, the significances of our study is that it contributes to literature: (1) identifying factors that contribute most to groundwater potential, (2) illustrating the effectiveness of ensemble modeling for groundwater potential, and (3) improving the training and validation performances of the base ANN up to 17 and 11%.

The advantages of such modeling studies for water resource management are: (1) delineating the landscapes in terms of groundwater potential, (2) strengthening of the decision-making process, (3) incorporating different stakeholders into the decision-making process, (4) suggesting an effective organizational framework for water consumptions, (5) developing monitoring systems for the protection of water resources, and (6) promoting water-saving agricultural facilities, and (7) reorganizing the industrial structure to compress the high water-consumption industries.

The present study is multidisciplinary approach, based on the algorithms used for the diagnosis in Medical and health field, thus new hybrid artificial intelligence approach developed in this study can also be used in the medical and health field with suitable modifications.

## Figures and Tables

**Table 1:** Factor ranks extracted using correlation-based feature selection method.

Rank	AM	Factor
1	0.31322	Elevation
2	0.26063	SPI
3	0.21741	STI
4	0.17321	River density
5	0.17194	Aspect
6	0.15272	Infiltration
7	0.07527	Landuse
8	0.07062	Slope
9	0.04683	Curvature
10	0.0371	TWI
11	0.02007	Soil
12	0.00438	Rainfall

AM: average merit; SPI: stream power index; STI: sediment transport index; TWI: topography wetness index.

**Table 2:** Training and validation performances of the models.

No	Index	Training		Validation	
		ANN	RABANN	ANN	RABANN
1	TP	22	26	8	8
2	TN	26	27	7	8
3	FP	5	1	6	5
4	FN	3	2	3	3
5	PPV (%)	81.48	96.30	57.14	61.54
6	NPV (%)	89.66	93.10	70.00	72.73
7	SST (%)	88.00	92.86	72.73	72.73
8	SPF (%)	83.87	96.43	53.85	61.54
9	ACC (%)	85.71	94.64	62.50	66.67
10	Kappa	0.713	0.893	0.260	0.338
11	RMSE	0.369	0.224	0.555	0.504

ANN: artificial neural network; RABANN: the ensemble model of RAB and ANN; TP: true positive; TN: true negative; FP: false positive; FN: false negative; PPV: positive predictive value; NPV: negative predictive value; SST: sensitivity; SPF: specificity; ACC: accuracy; RMSE: Root Mean Squared Error.
